# Nutritional rickets presenting with developmental regression: a rare presentation of rickets

**DOI:** 10.1186/s12887-023-04127-6

**Published:** 2023-06-29

**Authors:** Chariklia Pieridou, Suma Uday

**Affiliations:** 1 Department of Endocrinology and Diabetes, Birmingham Women’s and Children’s Hospital, Steelhouse Lane, Birmingham, UK; 2 Institute of Metabolism and Systems Research, University of Birmingham, Edgbaston, Birmingham, UK

**Keywords:** Rickets, Vitamin D deficiency, Developmental regression, Hypovitaminosis D, Milestones

## Abstract

Rickets is a disorder of defective mineralisation of the growth plate. Vitamin D deficiency remains the leading cause of nutritional rickets worldwide.

We present the case of a 3.5-year-old breastfed boy who presented with dental abscess when a history of developmental regression was noted. Clinical assessment revealed hypotonia, poor growth and stunting. Biochemistry identified hypocalcaemia (1.63mmol/L, [normal range (NR) 2.2-2.7mmol/L]), severe vitamin D deficiency (25hydroxyvitamin D 5.3nmol/L, [NR > 50nmol/L]) with secondary hyperparathyroidism (Parathormone 159pmol/L, [NR 1.6-7.5pmol/L]) and rickets on radiographs. Growth failure screening suggested hypopituitarism with central hypothyroidism and low IGF1 at baseline, however, dynamic tests confirmed normal axis. Management included nasogastric nutritional rehabilitation, cholecalciferol and calcium supplementation and physiotherapy. A good biochemical response in all parameters was observed within 3 weeks and reversal of developmental regression by 3 months from treatment. Developmental regression as a presentation of nutritional rickets is rare and requires a high index of suspicion.

## Introduction

Rickets is a disorder of defective chondrocyte differentiation and mineralisation of the growth plate [1] and hence only seen in growing children. Nutritional rickets remains the leading cause of rickets globally [2]. It is caused by vitamin D deficiency as a result of insufficient sunlight exposure or calcium deficiency secondary to poor dietary calcium intake, or a combination of the two. The resultant secondary hyperparathyroidism and subsequent hypophosphataemia leads to defective vascularisation and apoptosis of the terminal hypertrophic chondrocytes [3] and reduction in metaphyseal mineralisation.

Despite numerous preventative strategies [4], in the most recent decades the incidence of nutritional rickets has been observed to rise worldwide highlighting the major public health problem it is. In the United Kingdom, it is estimated that 1.39 per 100,000 children younger than the age of 5 years are annually diagnosed with the condition [5], a number undoubtedly underestimated [6]. A multitude of causes have been identified in line with the increase in prevalence of vitamin D deficiency. Vitamin D deficiency is defined by 25(OH)D levels below 30nmol/L and insufficiency by levels between 30 and 50nmol/L^4^. The risk is highest amongst dark-skinned populations resident in high latitude countries with restricted sunlight exposure [7], and poor supplementation [8]. Prolonged exclusive breastfeeding beyond 6 months of age is a well-established risk factor for deficiency [9].

The clinical features of nutritional rickets are both varied and potentially life-threatening [10] with implications persisting into adulthood. Children may present with seizures, paraesthesias, tetany [9] or cardiomyopathy [11, 12] as a result of hypocalcaemia. The defective bone mineralisation leads to softening of growing bones resulting in bone pain, bowed-leg or knock-knee deformities, muscular weakness, delayed motor development and stunted growth [10–13]. The diagnosis is made on the basis of history, clinical examination and biochemical findings and confirmed by the radiological findings of growth plate widening and metaphyseal cupping and fraying [4].

Rickets as a cause of developmental delay has been reported in a handful of cases in the literature [9, 14, 15]. Here we present a child with severe nutritional rickets presenting with developmental regression and features of hypopituitarism, a less common presentation.

## Case report

Presentation: A three-and-a-half-year-old boy was referred to the general paediatric team by the dentist, due to concerns regarding developmental regression, following admission for incision and drainage of his dental abscess. He experienced regression in his motor milestones; losing the ability to sit independently over the last twelve months and to walk over the last seven to eight months.

Birth history: The child was born in the UK (52°N), at term following normal vaginal delivery to non-consanguineous parents of Pakistani background, with a birth weight of 3.5 Kg and had an unremarkable post-natal course.

Past History: The child had no significant past medical history but was noted to have repeated contact with the health visitor due to longstanding feeding difficulties and reluctance to consume solid food. As a result, he did not achieve timely weaning and was almost exclusively breastfed at presentation. The child was reported to be on healthy start vitamins although he was not compliant with this. There was no reported bowing of his legs.

Development history: The child appropriately achieved his gross and fine motor and speech developmental milestones until the age of two and a half years. He was able to sit unsupported at 6 months of age and was walking independently at 12 months of age. There were no reported concerns in regards to his vision and hearing. At the time of review, he was communicating in short sentences and demonstrated fine motor and cognitive efficiency in navigating himself through electronic devices and playing video games.

Family history: Mother had chronic rheumatoid arthritis. Two older siblings had arthrogryposis.

**Fig. 1:**
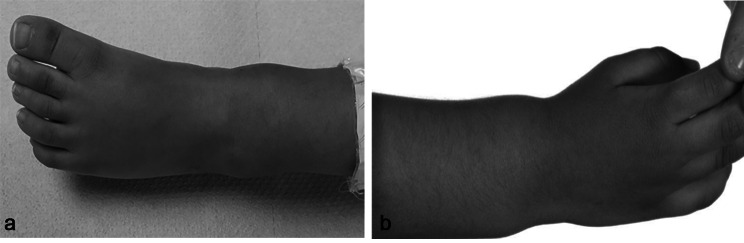
(**a**) Swollen ankle. (**b**) Swollen wrist

**Fig. 2:**
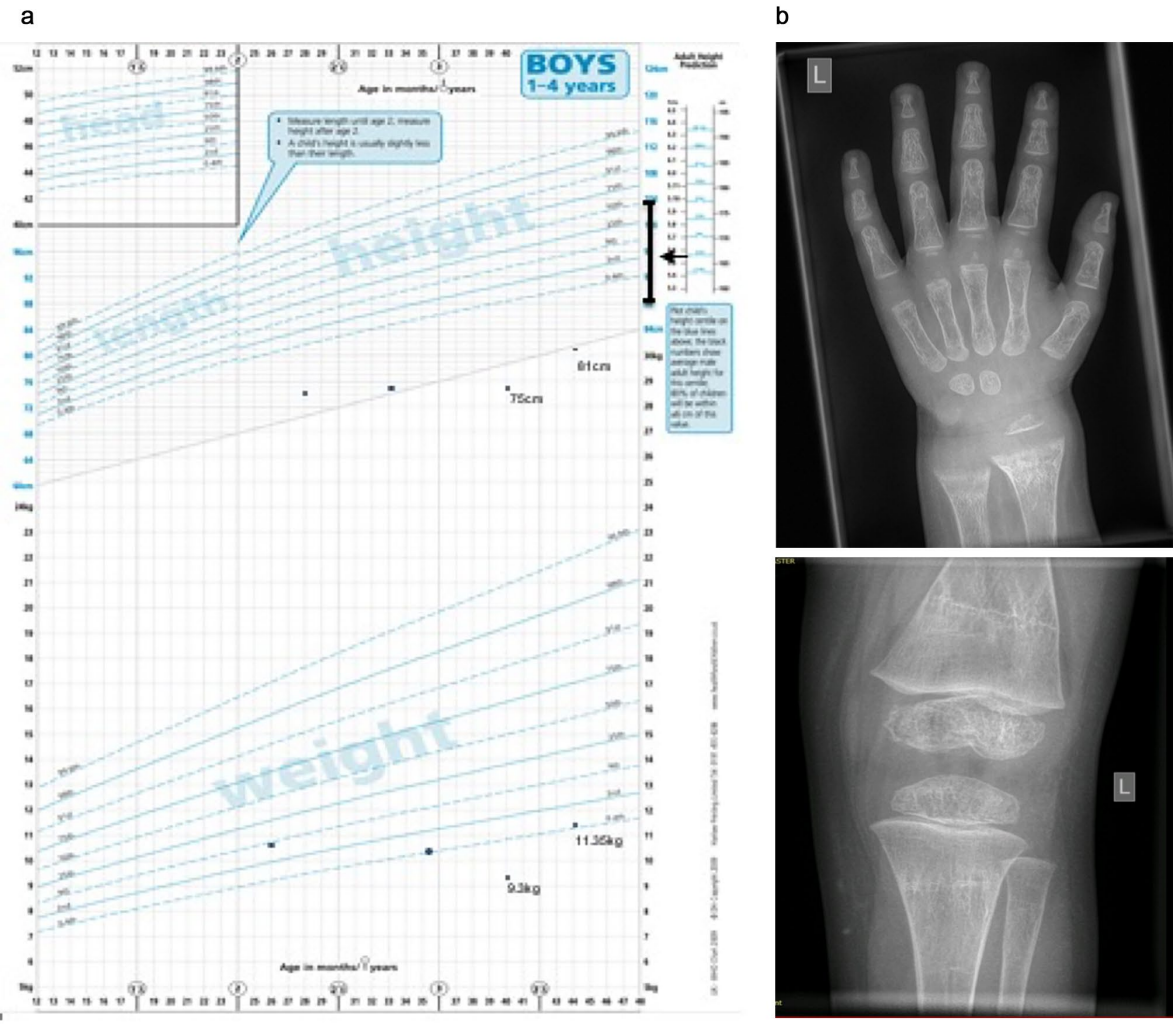
(**a**) Growth chart demonstrating growth over the preceding 18months and fall below Mid Parental Height centiles. (**b**) X-ray of wrist (left) before treatment and knee (right) after treatment showing improvement of metaphyseal fraying and cupping

Physical examination: Revealed suboptimal oral hygiene with dental carries, thin hair and bilateral swelling of his ankle (Fig. 1a) and wrist joints (Fig. 1b). He had truncal and peripheral hypotonia. There were no signs of wasting. At presentation he was able to sit without support but could not pull to stand or walk. He appeared severely stunted (height-for-age SDS − 5.99) and underweight (weight-for-age SDS − 3.88). A significant drop was noted in his linear growth for last twelve months and a noteworthy fall outside his mid-parental height (MPH) centile range (Fig. 2a). His weight for height SDS was adequate (weight-for-height SDS of -1).

Investigations: Initial biochemical profile (Table 1) revealed hypocalcaemia, hypophosphataemia, elevated alkaline phosphatase, elevated parathyroid hormone and low 25 hydroxy-vitamin D (25OHD) levels, suggestive of severe vitamin D deficiency. Rickets was confirmed on radiographs (Fig. 2b). Maternal investigation revealed severe vitamin D deficiency (25OHD < 7.5nmol/L).

**Table 1:** Summary of initial biochemical parameters

Parameter	Results	Normal range
Adjusted calcium	**1.63**	2.2–2.7 mmol/L
Phosphate	**0.7**	0.9–1.8 mmol/L
ALP	**1776**	80–330 IU/L
Urea	2.5	2.5–6.5 mmol/L
Creatinine	13	23–37 µmol/L
25(OH)D	**< 7.4 (5.3)**	> 50 nmol/L
Vitamin B12	**151**	283–1613 ng/L
Folate	15.6	> 11.9 µg/L
PTH	**159**	13-29 ng/L
Free Thyroxine	**8.3**	10.8–22.9 pmol/L
TSH	2.65	0.5–3.8 mU/L
IGF-1	**< 2**	3.6–14.8 nmol/L
IGF Binding Protein (3)	2.12	1.64–4.49 mg/L
FSH	0.4	0.2-3.0 U/L
LH	< 0.1	< 2.6 U/L
Cortisol^#^	0’ 239 30’ 871 60’ 961	Peak > 500 nmol/L
Growth Hormone*	0’ 8.4 30’ 2.5 60’ 5.3 90’ 4.2 120’ 2	Peak > 6.7 g/L

ALP; Alkaline phosphatase, 25(OH)D; 25 Hydroxy-vitamin D, PTH; Parathyroid Hormone, TSH; Thyroid Stimulating Hormone, IGF-1; Insulin-like Growth Factor-1, FSH; Follicle Stimulating Hormone, LH; Luteinising Hormone

^#^ Short Synacthen Test

* Arginine stimulation test

Initial blood investigations also revealed low levels of free thyroxine (T4) with inappropriately low TSH indicating central hypothyroidism. Additionally, a low insulin-growth factor 1 (IGF1), in the setting of severe stunting (Fig. 2a) led to further evaluation of the full endocrine axis for hypopituitarism. IGFBP3 done to evaluate nutritional vs. non-nutritional causes of stunting was normal. A sufficient peak in growth-hormone on arginine stimulation test and cortisol on short synacthen test were noted. Coeliac screen was negative. MRI brain was undertaken to evaluate the pituitary gland morphology and any additional neurological causes of developmental regression, which was normal. Similarly, the developmental regression led to screening for metabolic conditions with urine organic acids and amino acids, which did not yield any positive results.

Management: The child was started on cholecalciferol 6000 International Units daily for 12 weeks as per global consensus recommendations guidelines [4] and calcium supplements 500 mg daily. Calcium supplements was also continued for 12 weeks and then gradually weaned by incorporating calcium in diet over the next 6–8 weeks. Nutritional rehabilitation via nasogastric (NG) tube feeding and physical rehabilitation through physiotherapy input were initiated following admission. The effects of cholecalciferol and calcium treatment on the biochemical parameters are as shown in Table 2. On regular follow-up his developmental regression was observed to resolve. At discharge, 4 weeks from admission, the child was able to pull to stand. He was able to run when reviewed in the outpatient clinic at 3 months from treatment start. Gradual progress with oral feeds was made with speech and language therapy support. With continued nutritional rehabilitation, at the most recent follow up at 6 years of age his weight-for-age is at -2.5 SDS and height-for-age at -3.3 SDS which is within the parental target centile.

The negative findings on metabolic, endocrine and neurological evaluation suggest the nutritional aetiology of developmental regression which is confirmed with the positive response to cholecalciferol, calcium supplements and nutritional rehabilitation.

**Table 2:** Biochemical parameters at baseline and following treatment

Normal range	IGF-1 3.6–14.8 nmol/L	Free thyroxine or T_4_ 10.8–22.9 pmol/L	ALP 80–330 IU/L	Adjusted Ca 2.2–2.7 mmol/L	PO_4_ 0.9-1.8 mmol/L	PTH 13–29 ng/L
Baseline	< 2	8.2	1776	1.63	0.7	159
Week 2	6.5	13.1	-	-	-	-
Week 3	10.9	16	-	2.1	0.9	138
Week 5	-	-	1010	2.38	0.9	-
Week 8	-	-	711	2.37	1.3	-
Week 12	-	-	420			49

## Discussion

Developmental regression of motor milestones is a rare but serious presentation of severe vitamin D deficiency which is reversed on treatment. Severe nutritional deficiency manifesting as hypopituitarism is rare but prompt recovery with nutritional rehabilitation is noted.

Nutritional rickets is caused by vitamin D and/or dietary calcium deficiency. There are a handful of reports of children presenting with delayed motor development, in particular delay in walking as a result of nutritional rickets [9, 14, 15] however, reports of developmental regression are rare. Such presentations lead to exhaustive neurometabolic investigations which are time and resource consuming in addition to the distress and anxiety caused to families.

Given the limited vitamin D in diet, the main source remains skin synthesis, through the action of UVB on 7-dehydrocholesterol to produce cholecalciferol (vitamin D2) and ergocalciferol (vitamin D3) [16]. It is then activated in the liver and the kidney via two enzymatic hydroxylation reactions to a final product of calcitriol, the active form of vitamin D. Calcitriol is responsible for the supply of phosphate and calcium to the bone, allowing for mineralisation. Factors contributing to vitamin D deficiency in our patient were low exposure to sunlight due to high latitude residence and dark skin, prolonged breast feeding, lack of consumption of fortified foods which are limited and lack of adherence to supplementation [17]. The resultant reduced production of calcitriol decreases dietary calcium absorption from the gut leading to hypocalcaemia [16]. Moreover, with prolonged breastfeeding the calcium content in breast milk also reduces [18], predisposing the child to twin deficiencies exacerbating the pathology.

Breast milk is undoubtedly important for the infant; however, it is evident that prolonged exclusive breastfeeding is associated with malnutrition, short stature and poor weight gain [19, 20], owing to its low vitamin content [4, 18]. Beyond 6 months of age prolonged exclusive breastfeeding leads to vitamin D deficiency especially in the absence of maternal vitamin D sufficiency and/or adequate supplementation [20], as seen in our case (maternal 25(OH)D < 7.5nmol/L). It is therefore imperative that all women of childbearing age follow national recommendations for vitamin D supplementation; in the United Kingdom this constitutes a 400 IU/day supplement in all pregnant women and a 1000 IU/day dose in high-risk women [21].

Furthermore, there are existing concerns regarding the low levels of other micronutrients and vitamins, namely vitamins A and B in breastmilk [22]. A study of infants [n = 80] with birth weights between 2000 and 3000 g reported a negative linear association between duration of exclusive breastfeeding [n = 48] and both vitamin B levels (colabamin, riboflavin, but not folate) and gross motor development [21]. Similar to the above findings, we identified low serum B12 levels at baseline in our patient, with normal serum folate. The same results were not reported in formula fed infants [n = 32] or those exclusively breastfed for < 1 month [22]. Current recommendations from WHO suggest that weaning should start for most infants at 4–6 months. As infancy progresses the nutrient content of weaning foods becomes of increasing significance. Thus, feeding difficulties including failure in weaning, particularly when high-risk individuals are involved, warrant early referral to health care professionals, to allow for early recognition of nutritional deficiencies and prevention of severe disease.

Delayed walking as a presentation of nutritional rickets has been recognised. A study of children with confirmed vitamin-D deficiency rickets in Canada reported that 18% (10/56) of those aged 1–2 years had delayed walking [14], whilst another study of children aged 3–36 months from western Saudi Arabia reported delayed walking in 15% (9/60) [23]. In a study by Agarwal et al. [9], 59% (25/42) of children aged 1-2.9 years presenting with non or delayed walking were diagnosed with nutritional rickets. Of those, 100% showed radiological response to vitamin D and calcium supplementation by 2 weeks, with complete resolution averaging 5 months (range 2–8 months) and 100% biochemical resolution by 6 months. They further reported that 85% (17/20) of these children started walking within 3 months of treatment as noted in our patient.

Suspected hypopituitarism in the setting of severe stunting in our patient added to the burden of further evaluation of the pituitary axis. Numerous studies have previously hypothesised an interaction between vitamin D and IGF1/GH axis [24, 25], although it is yet to be elucidated how the two are intertwined. There is some evidence suggesting that vitamin D stimulates hepatic secretion of IGF-1 and IGF-1 receptor expression in several tissues [26]. However, low IGF-1 levels are linked to nutritional deficiency such as in patients with eating disorders with levels returning to normal upon weight restoration [27]. IGF-1 levels are closely linked to body fat and BMI [27] and recognised to be low in children with chronic illness such as cystic fibrosis, cyanotic congenital heart disease or short bowel syndrome [28]. It is therefore likely, that these changes were nutritional due to the child’s poor diet. Similar to IGF-1 levels, a reduction in thyroid hormones is also known to occur in states of chronic starvation, including anorexia nervosa, with reversal on refeeding [26]. In our patient, both IGF-1 and thyroxine levels normalised within 2 weeks of NG feeds, further supporting their link to nutritional depletion. Reduction in all anterior pituitary hormones including LH and FSH in nutritional depletion has been reported [26, 27], which was recorded in our patient. It is important to note that children with severe nutritional rickets may not necessarily appear malnourished or wasted [29].

The normal response of pituitary hormones (growth hormone, cortisol) on dynamic testing, normal brain imaging and the prompt normalisation of biochemical parameters following nutritional intervention supports nutritional deficiency as the main cause of presentation.

## Conclusion

Although severe vitamin D deficiency is known to cause developmental delay and myopathy, developmental regression as a presentation of nutritional rickets is rare. Clinical knowledge of such rare presentations and a high index of suspicion can prevent unnecessary clinical investigations. Prolonged breastfeeding is a recognised cause of vitamin D deficiency, especially in the absence of appropriate supplementation. Prompt supplementation and treatment with vitamin D and calcium can improve both the immediate and long-term outcomes.

## Data Availability

All data generated or analysed during this study are included in this published article.
